# Direct anterior versus posterolateral approaches for clinical outcomes after total hip arthroplasty: a systematic review and meta-analysis

**DOI:** 10.1186/s13018-020-01747-x

**Published:** 2020-06-23

**Authors:** Wang Chen, Jian-Ning Sun, Ye Zhang, Yu Zhang, Xiang-Yang Chen, Shuo Feng

**Affiliations:** Department of Orthopedic Surgery, Affiliated Hospital of Xuzhou Medical University, 99 Huaihai Road, Xuzhou, 221002 Jiangsu China

**Keywords:** Total hip arthroplasty, Direct anterior approach, Posterolateral approach

## Abstract

**Objective:**

The main objective of our study was to compare the intraoperative and postoperative outcomes of direct anterior approach (DAA) with posterolateral approaches (PLA).

**Methods:**

We searched Cochrane library, Web of Science, and PubMed for literatures comparing DAA with PLA. On the basis of inclusion and exclusion criteria, relevant literatures were selected. Two members independently screened qualified literatures, evaluated the literature quality, and extracted data information.

**Results:**

Eighteen randomized controlled trials (RCTs) and non-RCTs totaling 34,873 patients (DAA = 9636, PLA = 25237) were contained in this systematic review and meta-analysis. The results showed that DAA were reduced in terms of length of hospital stay (weighted mean difference (WMD) = −0.43, 95% confidence interval (CI) −0.78 to −0.09, *P* = 0.01), LLD (WMD = −2.00, 95% CI −2.75 to −1.25, *P* < 0.00001), PE/DVT (WMD = 0.36, 95% CI 0.15 to 0.85, *P* = 0.02), dislocation (WMD = 0.42, 95% CI 0.30 to 0.59, *P* < 0.00001) and visual analog scale (VAS) (WMD = −0.57, 95% CI −0.91 to −0.23, *P* = 0.0009) compared with PLA; however, DAA compared with the PLA was increasing in terms of operative time (WMD = 14.81, 95% CI 7.18 to 22.44, *P* = 0.0001), intraoperative blood loss (WMD = 105.13, 95% CI 25.35 to 184.90, *P* = 0.01), fracture (WMD = 1.46, 95% CI 1.00 to 2.11, *P* = 0.05), and Harris hip score (HHS) (WMD = 1.19, 95% CI 0.77 to 1.61, *P* < 0.00001).

**Conclusions:**

DAA was preferable effectiveness to PLA in early pain relief and functional recovery; however, PLA has a shorter operation time, intraoperative less blood loss and fracture.

**Trial registration:**

Registration ID, CRD42020151208

## Introduction

Total hip arthroplasty (THA) is a valid method for the treatment of hip diseases such as femoral neck fracture, aseptic necrosis of femoral head, developmental hip dysplasia, and rheumatoid arthritis [1]. It can significantly eliminate the patients’ hip pain, restore hip function, get rid of pain, and improve the quality of life [2]. During the 10-year follow-up, the clinical efficacy of THA has been significantly improved and the survival rate of the prosthesis exceeded 95% [3].

There are various approaches for THA: anterolateral approach (ALA), posterolateral approaches (PLA), direct anterior approach (DAA), direct lateral approach (DLA), etc. ALA is performed via the gluteus medius, vastus lateralis, and external rotators [4]. Static Trendelenburg test showed positive evidence, suggesting that ALA increases hip load and affected hip abduction. Posterior approach is the most widely applied total hip arthroplasty in the world [5]. The direct anterior approach does not cut off any muscle tissue around the hip, it does not cause any damage to the posterior joint capsule particularly, theoretically reducing the risk of dislocation [6, 7]. Registration data from the UK and New Zealand indicate that most primary THA operations are performed using PLA, and less than 5% of surgeons use DAA [8, 9]. Some scholars reported that patients who received PLA had higher postoperative levels of creatine kinase, a marker of muscle inflammation [10].

Alternative, less invasive approaches to total hip arthroplasty are attracting increasing attention [11]. Studies have shown that MIS-THA (little or no muscle dissection) can reduce soft tissue injury and blood loss, further promote postoperative recovery and accelerate the recovery of normal daily functions [12–14]. Surgeons can improve the DAA based on the gap between tensor fascia lata, sartorius, and rectus femoris muscle (Heuter gap) [15]. Relative to the conventional PLA, the method of DAA has the benefits of less bleeding, shorter duration of pain, shorter length of hospital stay, and a lower rate of hip dislocation. On the contrary, many of the literatures have made clear that two types of THA have similar prognosis in the early postoperative period; however, the incidence of complications in DAA is relatively high, especially during early technical learning stages [16–19].

Several more high-quality RCT [20–23] and non-RCTs [24–27] have been published without conclusive results. Although these literatures hold many new views, it is necessary to analyze this issue because of their single demonstration, a one-sided focus on clinical results, lack of latest data, and lack of recommendation strength. Jia et al. [28] performed a meta-analysis, he compared the two approaches of DAA and PA. However, PA includes PLA, so his inclusion criteria were wider and biased. Wang et al. [29] analyzed the results of DAA and LA, nevertheless, LA is not PLA. Although they conclude that DAA has good results in many aspects, the results of DAA compared with PLA require further study.

## Methods

Our study was based on the PRISMA guidelines (the preferred reporting item for systematic review and meta-analysis) [30]. This meta-analysis was prospectively registered with Prospero International prospective register of systematic reviews (www.crd.york.ac.uk/prospero/) (Registration ID: CRD42020151208).

### Literature search

In this systematic review and meta-analysis, we searched Cochrane Library, Web of Science, and PubMed for comparative studies of DAA and PLA. With the following search terms: (1) “total hip arthroplasty (All Fields)” OR “total hip replacement (All Fields)” OR “THA (All Fields)”; (2) “direct anterior approach (All Fields)” OR “DAA (All Fields)”; (3) “posterolateral approach (All Fields)” OR “PLA (All Fields)” OR “posterior approach (All Fields)” OR “PA (All Fields)”; (4) (1) AND (2) AND (3). We got the literatures we need by reading the title, abstract, and complete manuscript. We stipulated that the search of the literatures were not restricted by language. Reference list of relevant meta-analyses was queried to obtain studies that might have been missed.

### Inclusion criteria

Inclusion criteria for this meta-analysis followed the participant, intervention, comparison, and outcomes (PICO) approach.

1. Participant: patients who were willing to undergo primary total hip arthroplasty

2. Intervention: patients who underwent direct anterior approach

3. Comparison: patients who underwent posterolateral approach

4. Outcomes: we stipulated that inclusion in the literatures should include at least any of the following results. (1) Primary outcomes: HHS (Harris hip score), VAS (visual analog scale), HOOS (hip disability and osteoarthritis outcome), operation time, intraoperative blood loss, length of hospital stay; (2) secondary outcomes: incision length, fracture, infection, PE/DVT (pulmonary embolism/deep vein thrombosis), pneumonia, dislocation, hematoma, LLD (leg-length difference), re-operation, abduction angle, anteversion angle

5. Study design: RCT or high-quality non-RCT

### Exclusion criteria

1. Simple studies of methods for DAA or PLA without comparative analysis

2. No relevant literature data information

3. Repeated reports and reviews

### Study selection

Two professionally trained researchers (YZ, JNS) independently screened the literatures and extracted the information. In case of any disagreement, discuss or submit it to a third party (YZ) who has received professional training. We expurgated duplicate literatures using the delete option of the software Endnote X9. On the basis of the criteria, we picked the studies out we needed and acquired the full text to extract the data, then read the title and abstract of the literatures to exclude the literature that did not match the study subjects, study type, and intervention measures. Literatures accorded with the inclusion criteria were further read literature content, excluding those that were repeatedly published, with incomplete data and poor credibility.

### Data extraction

Data from literatures were independently drawn by two investigators. By discussing with third parties, we resolved the extraction differences between the two researchers. The indexes extracted by the two researchers included basic information (author, country, sample size, age, BMI, study type, follow-up), score (HHS, VAS, HOOS), operation time, intraoperative blood loss, length of hospital stay, incision length, re-operation, complications (fracture, infection, PE/DVT, pneumonia, dislocation, haematoma), LLD, radiographic outcome (abduction angle, anteversion angle).

### Assessment of methodological quality

Cochrane risk assessment criteria were applied to estimate the literatures quality of the included RCTs: selection methods of the case group and control group, comparability between groups and exposure assessment methods; the literatures quality of non-RCTs were evaluated putting the Newcastle-Ottawa scale (NOS) into use, with a full score of 9, ≥ 7 for high quality literature, 5 to 6 for medium quality literature, and < 5 for low quality literature.

### Statistical analysis

Review Manager 5.3 was adopted for the analysis of included literatures data and a *P* value of < 0.05 in the data were defined as statistically significant. Dichotomous variables were applied using the odds ratio (OR) and 95% confidence interval (CI), while continuous variables were applied using weighted mean difference (WMD) and 95% CI. Performed a sensitivity analysis by deleting one study every time and rebuilding the data from the remaining studies to identify possible high heterogeneity studies. Funnel plot was drawn to test whether there was deviation in the included literatures.

## Results

### Study characteristics and quality evaluation

**Fig. 1:**
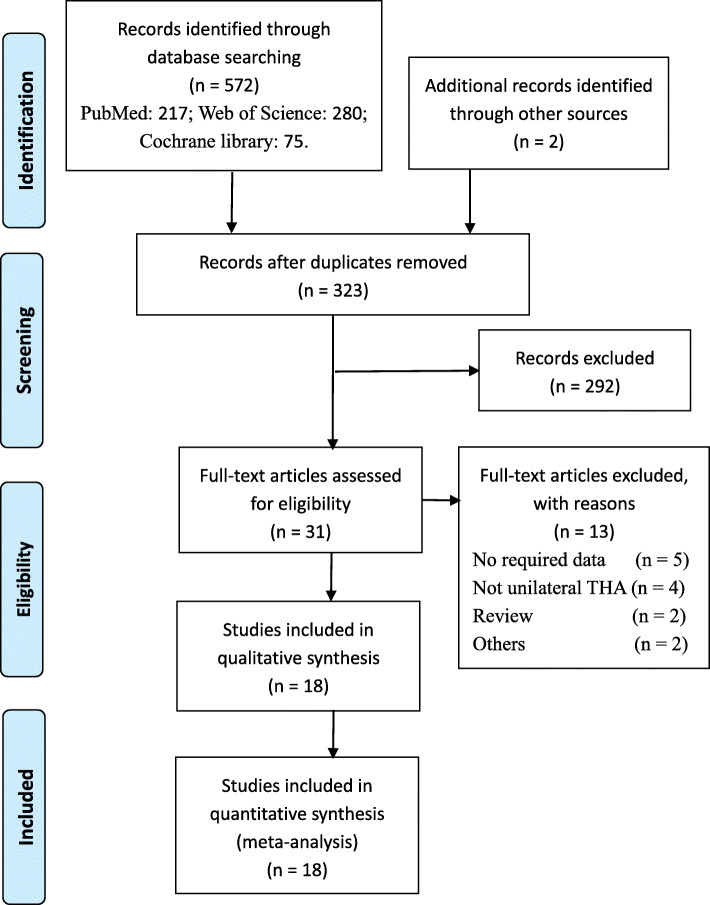
The flow diagram of literature selection

A total of 572 literatures were retrieved according to the search term. After deleting the duplicates using the software Endnote X9, 323 remained. Based on the specified inclusion and exclusion criteria, 292 articles were precluded. We perused the remaining 31 articles, 2 of which were systematic reviews. We excluded another 13 articles for the following reasons: no required data (*n* = 5); not unilateral THA (*n* = 4); review (*n* = 2); others (*n* = 2). Finally, this meta-analysis totally absorbed 18 RCT and non-RCTs. Literatures choice process was shown in Fig. 1.

Table 1 lists in detail the general characteristics of the studies after the final selection. Eighteen studies totaling 34,848 patients (DAA = 9624, PLA = 25,224) were contained. Four RCTs were low risk evaluated by Cochrane Collaboration risk of bias assessment tool, four prospective and ten retrospective cohort studies were high or middle quality evaluated by the Newcastle-Ottawa scale risk of bias assessment tool. We included relevant studies from 2012 to 2020. The sample size in the literatures were at least 25, at most 20,035. The follow-up time varies from 1.5 to 60 months.

**Table 1:** General characteristic of the included studies

Author/year	Country	DAA/PLA				Study	Follow-up	Outcomes
		Mean age (years)	No. of patients (*n*)	Female	BMI			
Hu [24]	China	58.2/59.3	110/98	68/58	25.8/25.6	Prospective	NC	5, 7, 10, 13, 14
Siljander [31]	USA	65/64	1846/3162	1002/1799	27.4/30.2	Retrospective	3	1, 4, 5, 6, 7, 10, 12
Wu [32]	China	49.67/48.21	24/23	9/10	22.16/23.32	Retrospective	15	1, 2, 3, 4, 13, 14, 15
Barrett [20]	USA	NC	43/44	NC	NC	RCT	60	15, 17
Daas [33]	Netherlands	74.8/72.1	41/26	33/22	27.6/28.1	Retrospective	12	17
Fleischman [34]	USA	62.7/66.6	5465/2160	2815/1164	28.4/28.8	Retrospective	24	5, 7
Godoy-Monzon [25]	Argentina	56.1/57.2	40/40	17/15	NC	Prospective	17.6	1,4,9,15
Triantafyllopoulos [35]	USA	62.3/64.2	1182/18853	626/10126	NC	Retrospective	50	3
Lee [36]	Korea	65.4/68.4	12/13	NC	NC	Retrospective	1.5	1, 2, 9
Rykov [21]	Netherlands	62.8/60.2	23/23	15/12	29/29.3	RCT	1.5	1, 2, 3, 15, 17
Zhao [22]	China	64.88/62.18	60/60	36/34	24.35/25.58	RCT	6	1, 2, 3, 4, 5, 13, 14, 15, 16
Fransen [37]	Netherlands	64.2/62.6	45/38	30/24	25/27.6	Retrospective	12	1, 2, 3, 14
Jelsma [26]	Netherlands	66.7/67.9	87/32	48/16	26.6/28	Prospective	6	3, 5, 6, 7, 8, 10, 11, 16, 17
Langlois [27]	France	86/85	38/44	32/19	21/23	Prospective	1.5	1, 3, 7, 9
Amlie [38]	Norway	67/66	421/421	291/268	NC	Prospective	36	7, 12
Barrett [23]	USA	61.4/63.2	43/44	14/25	30.7/29.1	RCT	12	1,2,4,5,7,8,13,14,15,16,17
Nam [39]	USA	66.76/66.86	110/110	71/65	28.3/27.4	Retrospective	6	13, 14
Spaans [40]	Netherlands	69/68	46/46	22/32	25/29	Retrospective	12	1, 2, 3, 5, 7, 9, 11, 14

*NC* not clear

1. operative time; 2. intraoperative blood loss; 3. length of hospital stay; 4. incision length; 5. fracture; 6. infection; 7. dislocation; 8. hematoma; 9. LLD (leg-length difference); 10. PE/DVT (pulmonary embolism/deep vein thrombosis); 11. pneumonia; 12. re-operation; 13. anteversion angle. 14. abduction angle; 15. HHS (Harris hip score); 16. VAS (visual analog scale); 17. HOOS (hip disability and osteoarthritis outcome)

### Risk of bias

**Fig. 2:**
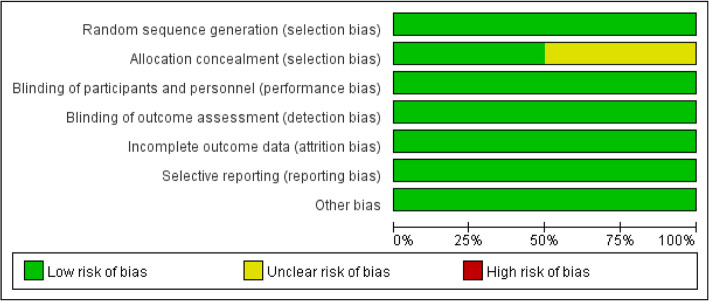
Risk of bias graph: review authors’ judgments about each risk of bias item presented as percentages across all included studies

**Fig. 3:**
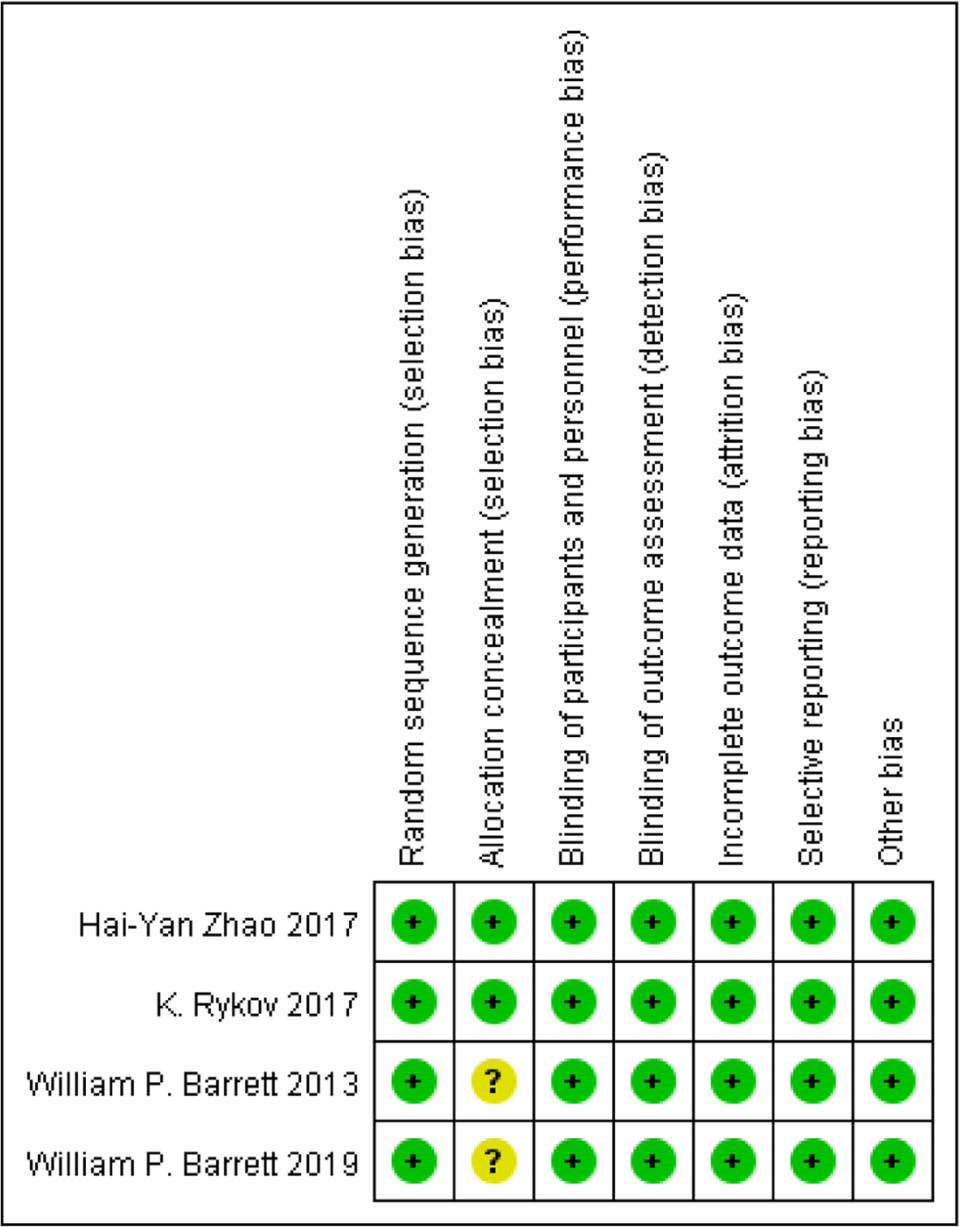
Risk of bias summary for included studies. +, no bias; −, bias; ?, bias unknown

Risk of bias graph and risk of bias summary of the randomized controlled study literatures are apart shown in Figs. 2 and 3. Four of these studies described the generation of random sequences, performance, and detection bias. Two studies described allocation concealment. Attrition bias, reporting bias, and other biases in the literatures were reported in detail, and these were classified as low-risk deviations. The literatures quality of the cohort study was appraised using the Newcastle-Ottawa scale. Table 2 presents the risk of assessing quality bias in the methodology of non-randomized controlled trials, and the results showed that the risk of bias was relatively low.

**Table 2:** The literatures quality of cohort studies were evaluated using Newcastle-Ottawa scale

Author/year	Selection	Comparability	Outcome	Total	Rating
Hu et al.	3	2	3	8	Excellent
Siljander et al.	3	2	3	8	Excellent
Wu et al.	3	0	3	6	Good
Daas et al.	3	2	3	8	Excellent
Fleischman et al.	3	2	3	8	Excellent
Godoy-Monzon et al.	4	1	3	8	Excellent
Triantafyllopoulos et al.	3	2	3	8	Excellent
Lee et al.	3	1	3	7	Good
Fransen et al.	3	2	3	8	Excellent
Jelsma et al.	4	2	3	9	Excellent
Langlois et al.	4	2	3	9	Excellent
Amlie et al.	4	2	3	9	Excellent
Nam et al.	3	2	3	8	Excellent
Spaans et al.	3	1	3	7	Good

### Outcomes

#### Operative time

**Fig. 4:**
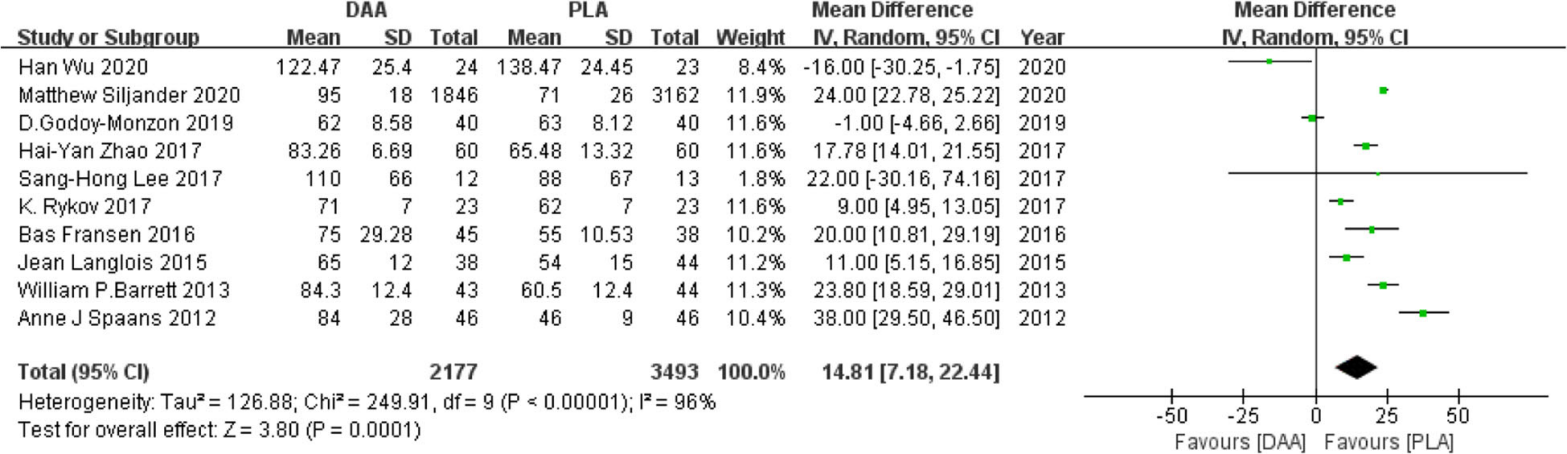
Forest plot comparing the operative time of DAA and PLA

**Fig. 5:**
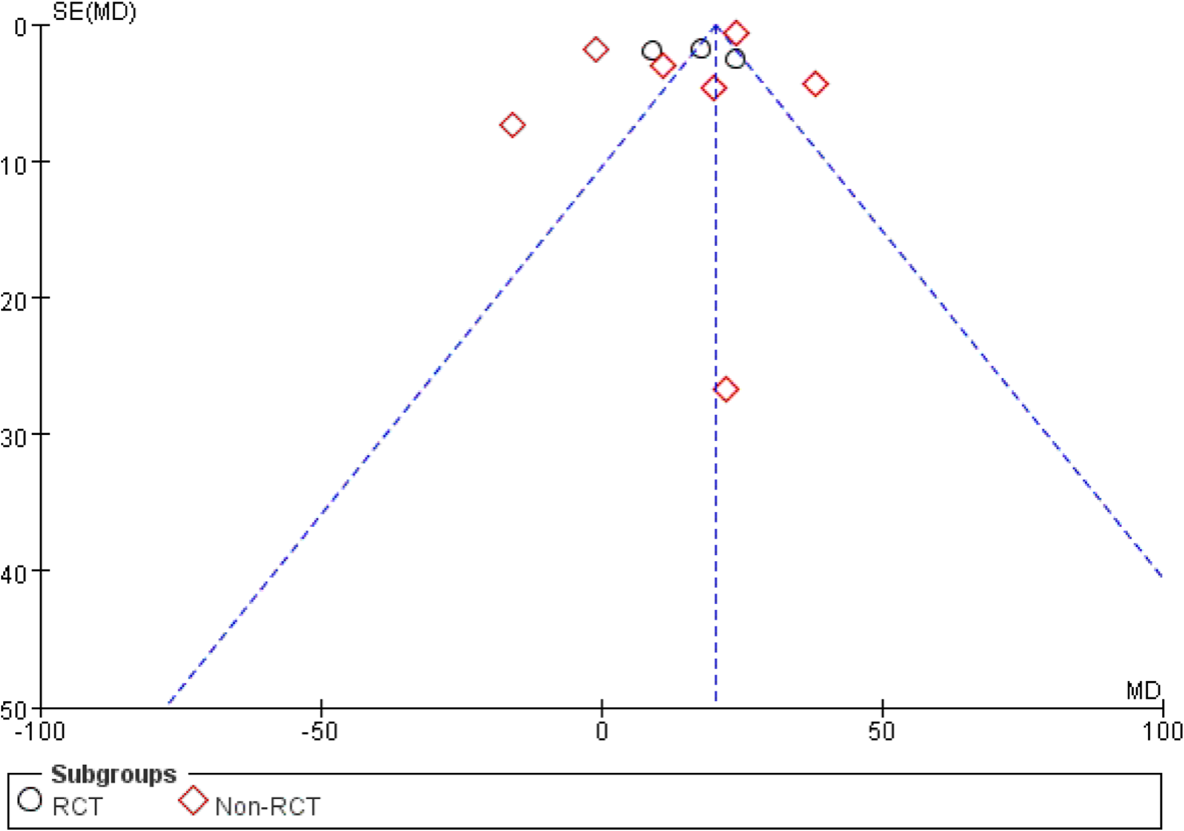
Funnel plots used to describe heterogeneity between RCT and non-RCT

Ten studies [21, 22, 23, 25, 27, 31, 32, 36, 37, 40] (including 5670 participants) reported a comparison of operative time between the two approaches, and the results obtained can prove that the operation time of PLA was less than that of DDA (Fig. 4). This project involved 10 of the 18 studies. We conducted a subgroup analysis and divided it into RCT and non-RCTs. As shown in the funnel plots, studies were basically symmetrical, indicating a small deviation (Fig. 5).

#### Intraoperative blood loss

**Fig. 6:**
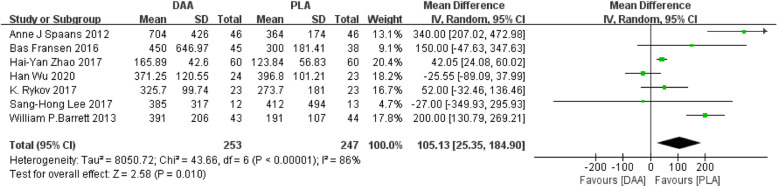
Forest plot comparing the intraoperative blood loss of DAA and PLA

Seven studies [21–23, 32, 36, 37, 40] (500 participants in total) recorded intraoperative blood loss for both surgical approaches, and the results obtained can prove that the intraoperative blood loss of PLA was less than that of DAA (Fig. 6).

#### Length of hospital stay

**Fig. 7:**
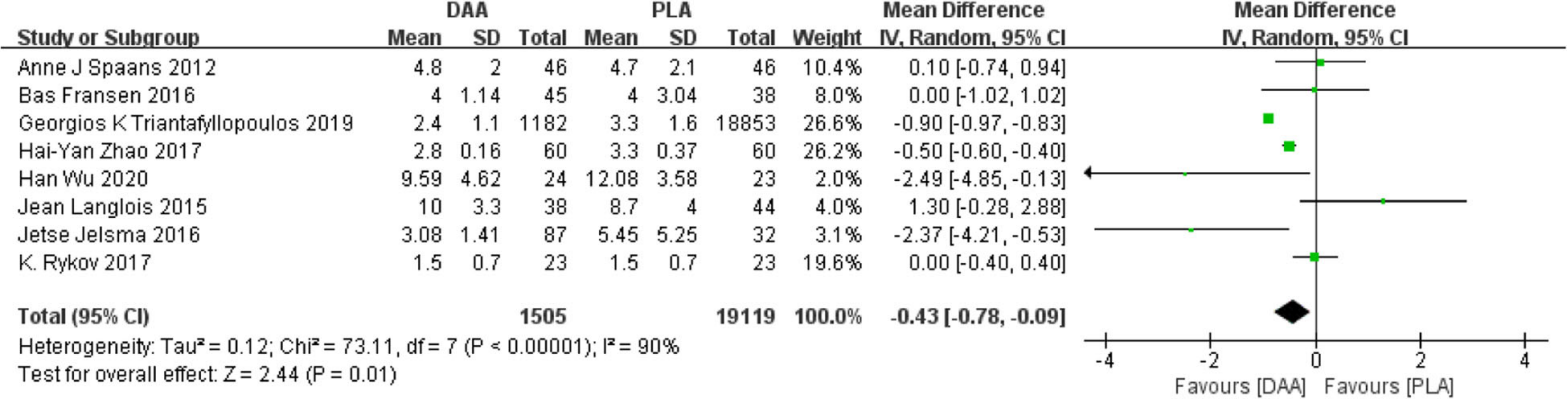
Forest plot comparing the length of hospital stay of DAA and PLA

Eight studies [21, 22, 26, 27, 32, 35, 37, 40] (20623 participants) reported a comparison of length of hospital stay after THA. The results obtained can prove that DAA had a shorter length of hospital stay (Fig. 7).

#### Incision length

**Fig. 8:**
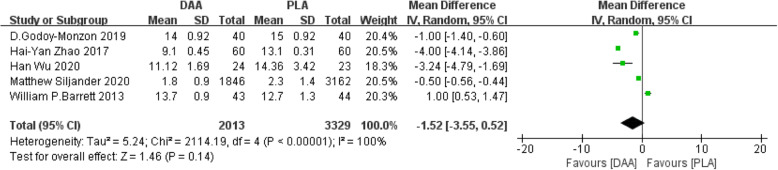
Forest plot comparing the incision length of DAA and PLA

Five studies [22, 23, 25, 31, 32] (5342 participants) reported a comparison of incision length after two types of surgery. The results obtained can prove that there was no significant difference in incision length after the two surgeries (WMD = −1.52, 95% CI −3.55 to 0.52, *P* = 0.14, Fig. 8).

#### Fracture

**Fig. 9:**
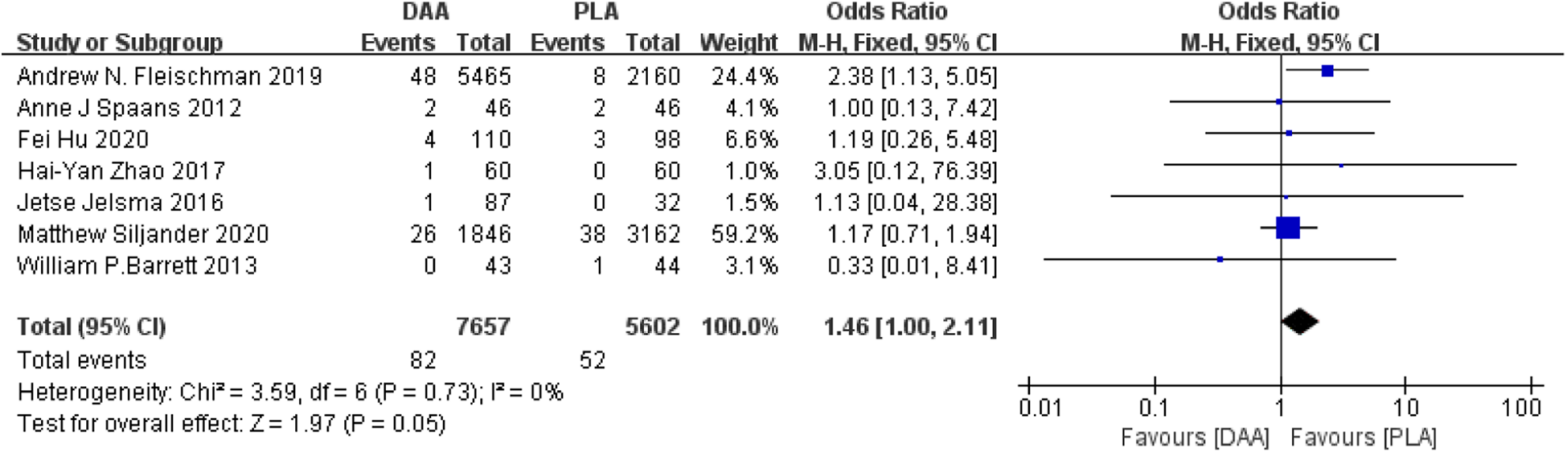
Forest plot comparing the fracture of DAA and PLA

Seven studies [22–24, 26, 31, 34, 40] (13,259 participants) reported a comparison of the incidence of fractures after the two surgeries, and the results obtained can prove that PLA has a higher fracture rate in patients than DAA (Fig. 9).

#### Infection

**Fig. 10:**
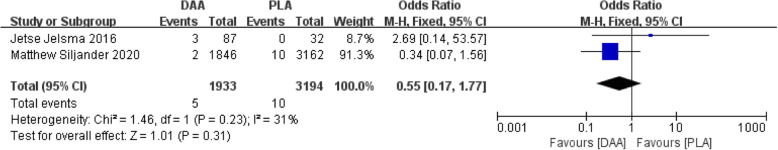
Forest plot comparing the infection of DAA and PLA

Two studies [26, 31] (5127 participants) reported a comparison of infection rates after the two surgeries, and results obtained can prove that there was no significant difference in infection rate between the two operations (OR = 0.55, 95% CI 0.17 to 1.77, *P* = 0.31, Fig. 10).

#### Dislocation

**Fig. 11:**
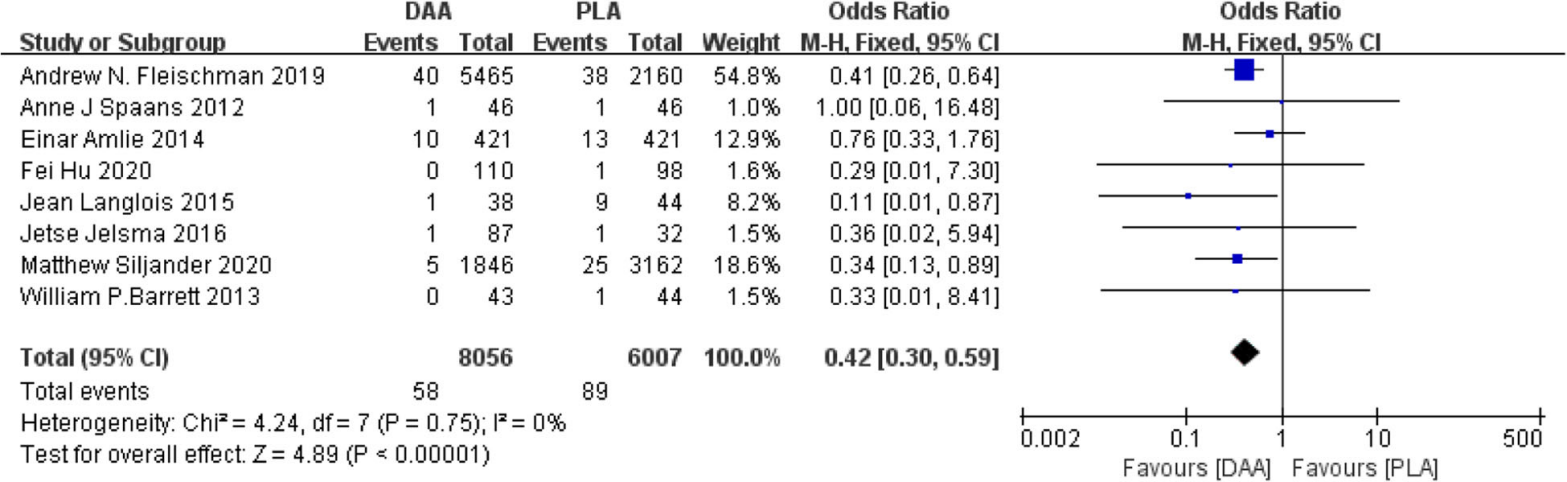
Forest plot comparing the dislocation of DAA and PLA

Eighteen studies [23, 24, 26, 27, 31, 34, 38, 40] (14,063 participants) reported a comparison of hip dislocation after two types of surgery, showing PLA has a lower hip dislocation rate than DAA (Fig. 11).

#### Hematoma

**Fig. 12:**
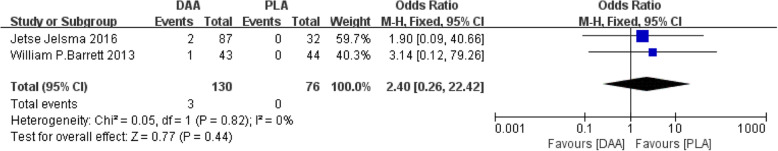
Forest plot comparing the hematoma of DAA and PLA

Two studies [23, 26] (206 participants) reported a comparison of the hematoma rate after the two surgeries. The results obtained can prove that the difference was no statistically significant (OR = 2.40, 95% CI 0.26 to 22.42, *P* = 0.44, Fig. 12).

#### LLD

**Fig. 13:**
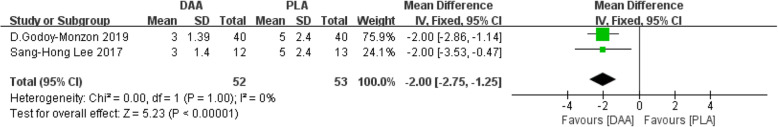
Forest plot comparing the LLD of DAA and PLA

Two studies [25, 36] (105 participants) reported a comparison of LLD between the two surgeries. Results showed that LLD after DAA surgery was less than PLA surgery (Fig. 13).

#### PE/DVT

**Fig. 14:**
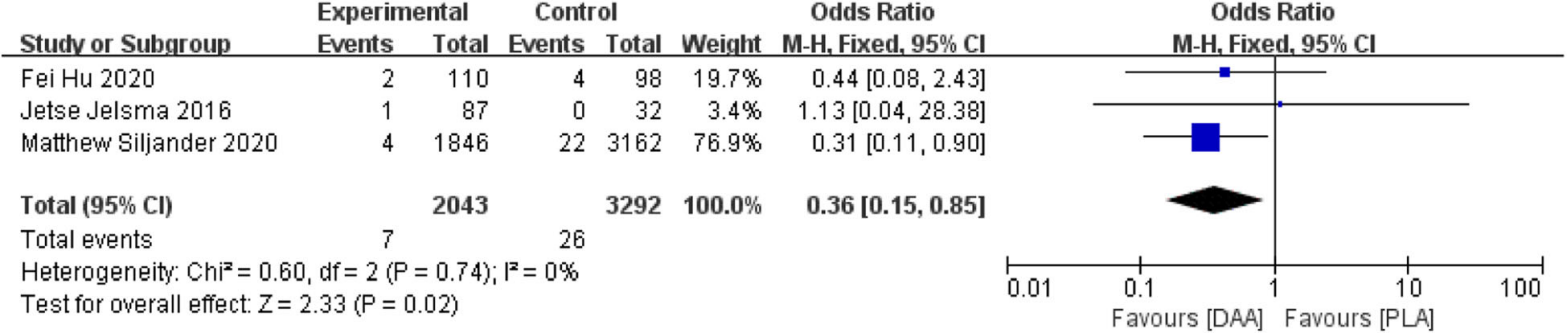
Forest plot comparing the PE/DVT of DAA and PLA

Three studies [24, 26, 31] (5235 participants) reported a comparison of PE/DVT between the two surgeries. Results showed that PE/DVT after DAA surgery was less than PLA surgery (Fig. 14).

#### Pneumonia

**Fig. 15:**
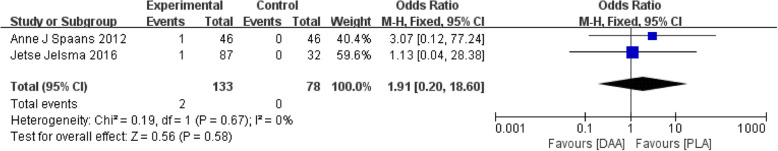
Forest plot comparing the pneumonia of DAA and PLA

Two studies [26, 40] (211 participants) reported a comparison of pneumonia between the two surgeries. Results showed that there was no significant difference in pneumonia (OR = 1.91, 95% CI 0.20 to 18.60, *P* = 0.58, Fig. 15).

#### Re-operation

**Fig. 16:**
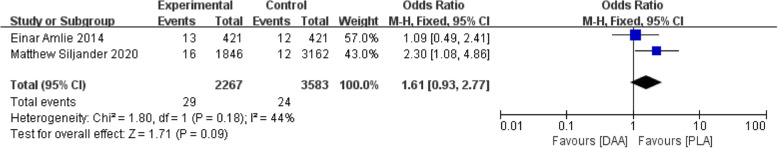
Forest plot comparing the re-operation of DAA and PLA

Two studies [31, 38] (with a total of 5850 participants) reported a comparison of the incidence of re-operation after the two surgeries, and the results obtained can prove that there was no significant difference in the incidence of re-operation (OR = 1.61, 95% CI 0.93 to 2.77, *P* = 0.09, Fig. 16).

#### Anteversion angle

**Fig. 17:**
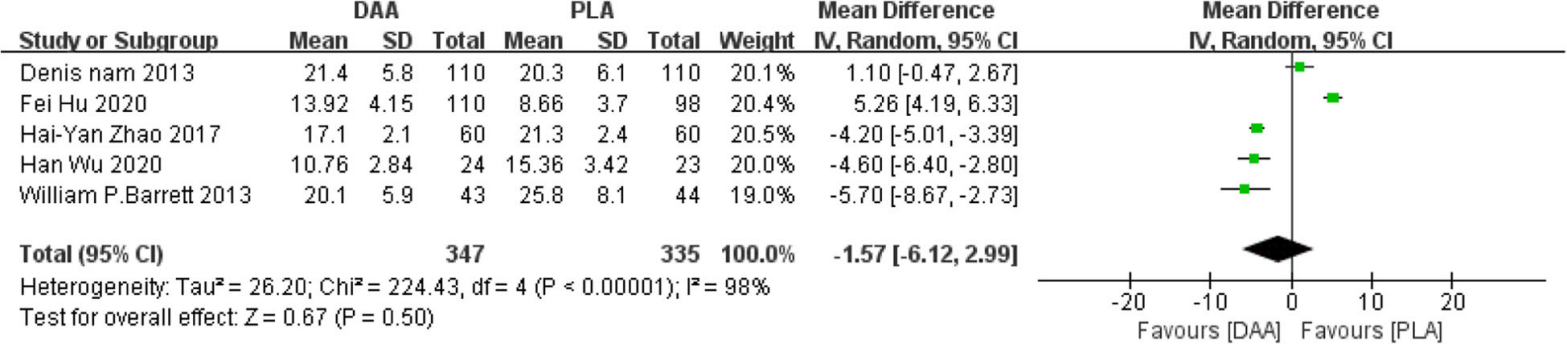
Forest plot comparing the anteversion angle of DAA and PLA

Five studies [22–24, 32, 39] (682 participants) reported a comparison of the anteversion angle after two types of surgery. The results obtained can prove that there was no statistically significant difference between the two postoperative anteversion angles (WMD = −1.57, 95% CI −6.12 to 2.99, *P* = 0.50, Fig. 17).

#### Abduction angle

**Fig. 18:**
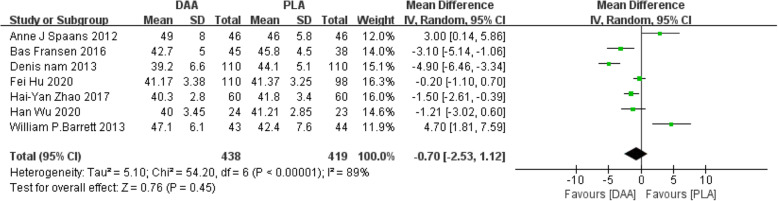
Forest plot comparing the abduction angle of DAA and PLA

Seven studies [22–24, 32, 37, 39, 40] (857 participants) reported a comparison of the abduction angle after two types of surgery. The results obtained can prove that there was no statistically significant difference between the two postoperative abduction angles (WMD = −0.70, 95% CI −2.53 to 1.12, *P* = 0.45, Fig. 18).

#### HHS

**Fig. 19:**
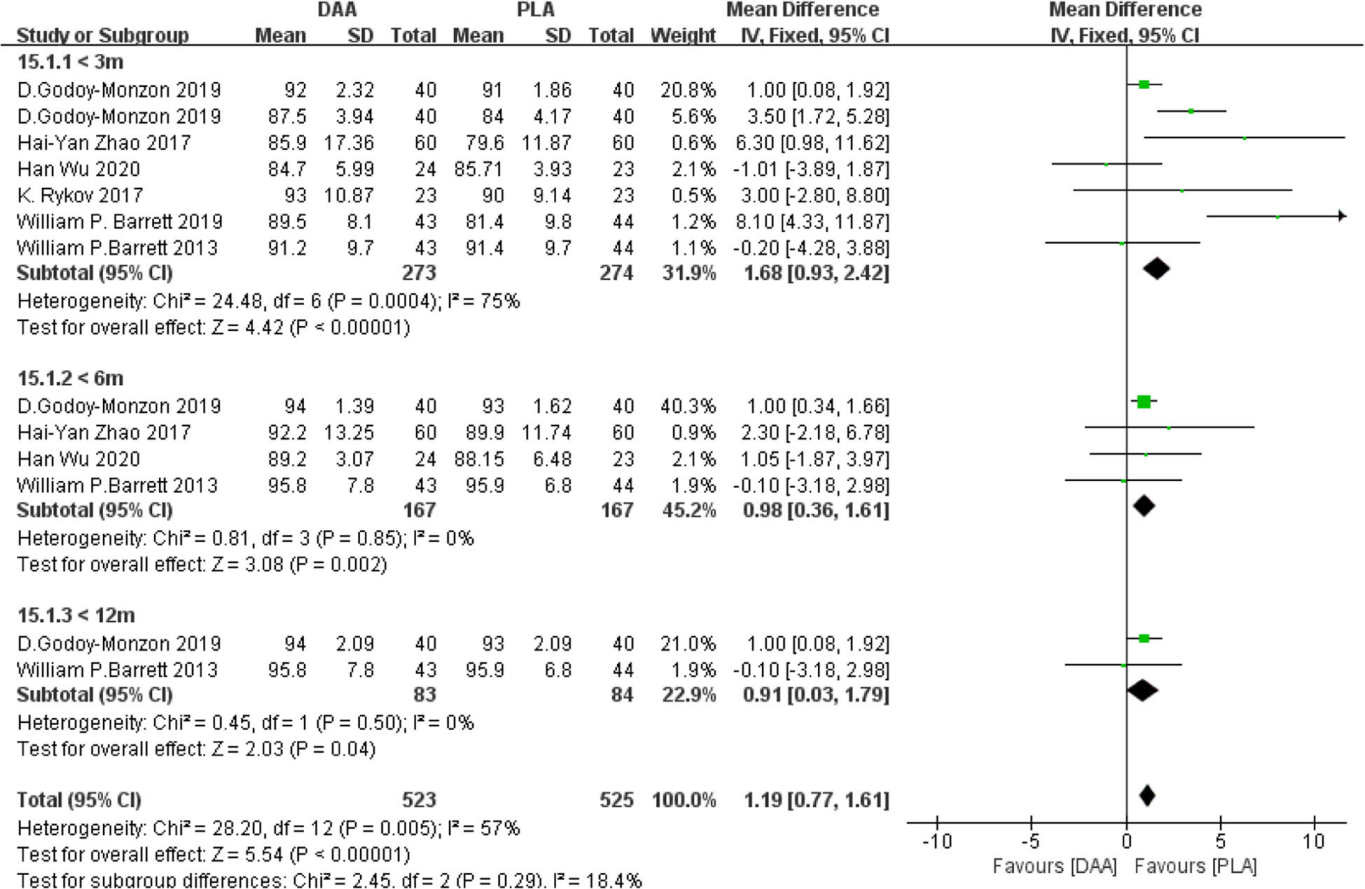
Forest plot comparing the HHS of DAA and PLA

Harris hip score (HHS) [20–23, 25, 32] was divided into three subgroups (< 3 m, < 6 m, < 12 m) according to the evaluation time. Six studies (1048 participants) were contained. The results indicated that the three periods of HHS of DAA were superior to that of PLA (Fig. 19).

#### VAS

**Fig. 20:**
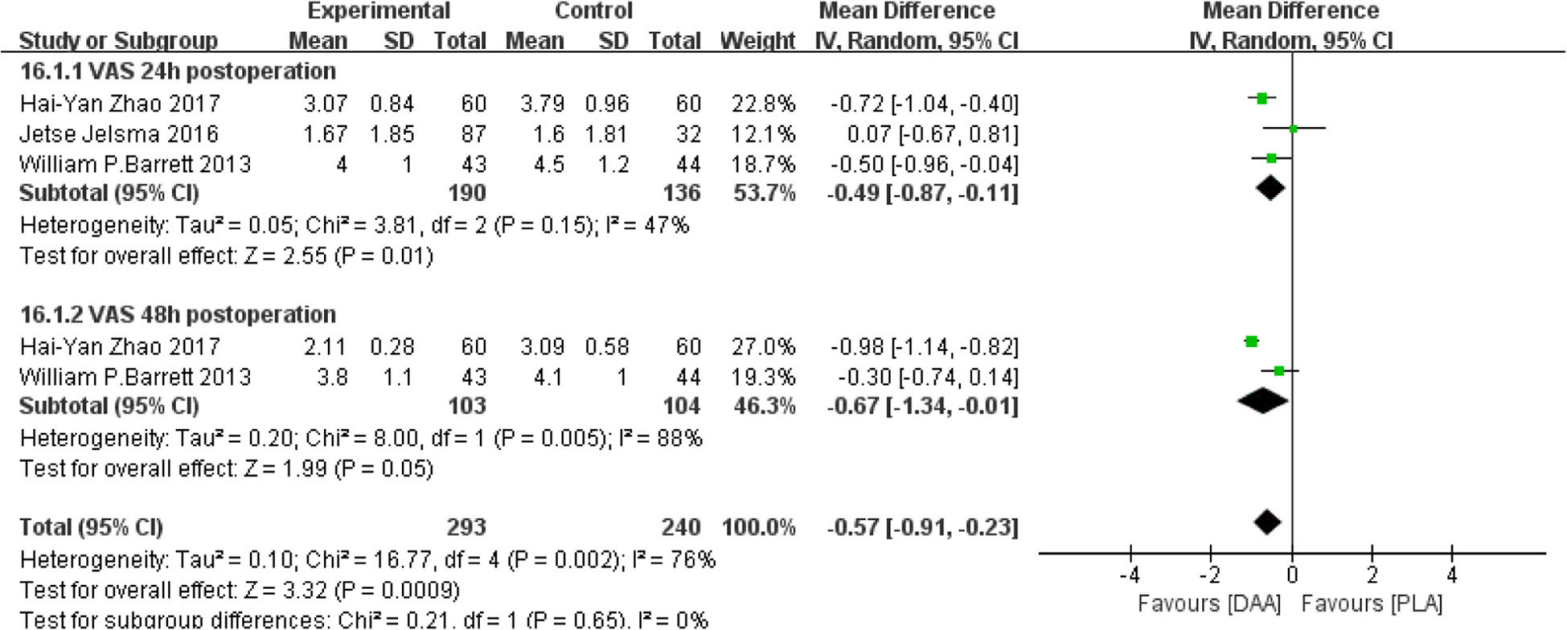
Forest plot comparing the VAS of DAA and PLA

Visual analog scale (VAS) [22, 23, 26] was divided into two subgroups according to time: VAS at 24 and 48 h. Three studies (533 participants) were contained. The results obtained can prove that the pain was lighter and relieved faster than that of PLA in 24 or 48 h after DAA (Fig. 20).

#### HOOS

**Fig. 21:**
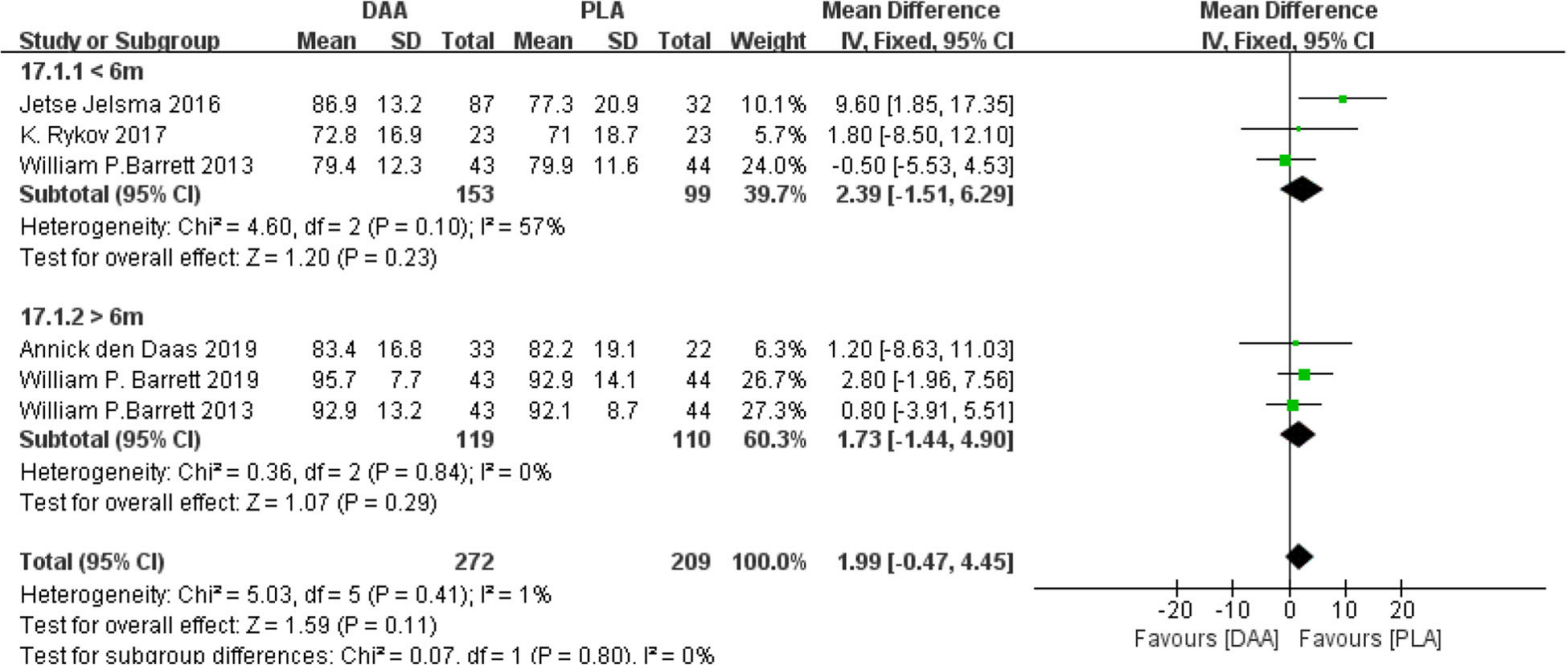
Forest plot comparing the HOOS of DAA and PLA

Hip disability and osteoarthritis outcome (HOOS) [20, 21, 23, 26, 33] was divided into two subgroups: mental composite scale and physical composite scale. Five studies (481 participants) were included. The results obtained can prove that the difference was no statistically significant (WMD = 1.99, 95% CI −0.47 to 4.45, *P* = 0.11, Fig. 21).

## Discussion

### Main findings

The results of our meta-analysis were sufficient to prove that, compared with PLA surgery, DAA had a shorter length of hospital stay and LLD, less PE/DVT and dislocation, faster and earlier recovery of hip function; however, DAA also had longer operative time and more intraoperative blood loss and fracture. There was no statistically significant difference between two groups in the aspects of incision length, pneumonia, infection, hematoma rate, re-operation. Postoperative imaging evaluation indicated that there was no statistically significant difference in abduction and anteversion angle.

### Comparison with previously published meta-analyses

There are many differences between us and the previously published meta-analysis. The article of Jia et al. [28] was an analysis of the PA, which includes the PLA. Since PA was not completely equal to PLA, the result of Jia et al. were different from the result of comparing DAA with PLA alone. Both Jia et al. and our study concluded that DAA had shorter hospital stays and longer operative times, and more fractures. Jia et al. concluded that there was no statistical difference between DAA and PLA in LLD and dislocation, while we believed that DAA had smaller LLD and less dislocation than PLA. Jia et al. focuses on the analysis of radiographic results, while we are more interested in intraoperative and post-operative clinical studies. Wang’s [29] analysis of the differences between DAA and lateral approach (LA) were fundamentally different from ours. Wang et al. compared DAA with LA, he believes that DAA is more beneficial than LA in reducing postoperative pain, blood loss, and increasing hip function. Our study found that DAA has more blood loss than PLA, which may be related to the longer operative time of DAA.

### Implications for clinical practice

Studies of Jewett and De Geest have shown that the incidence of complications of DAA surgery in the learning curve of surgeons is higher than that of PLA surgery, while the incidence of complications of DAA surgery after the learning curve of surgeons is lower than that of PLA surgery [41, 42]. In our meta-analysis, we selected literatures that specifically mentioned the surgeon’s surgery with high proficiency to discuss the occurrence of six complications. The results of our study prove that although there was no statistically significant difference in the three of these complications (pneumonia, infection, hematoma) between the two surgeries, the incidence of PE/DVT, dislocation after DAA was lower than that of PLA surgery. Compared with PLA, DAA has a shorter hospital stay, shorter LLD, less PE/DVT, hip dislocation, and early recovery of joint function. From these perspectives, DAA may be a better surgical method.

### Innovation of our meta-analysis

RCTs and high-quality cohort studies [20–27] were included in our study, and the literatures quality were relatively high. The majority of surgeons in the literature we included passed the learning curve, which made the results more reliable (Table 3). The literatures of inclusion were evaluated strictly, and the possibility of bias is small. The study has a large amount of data and a large number of participants, which is more credible.

**Table 3:** Whether the surgeon has passed the learning curve

Author	Surgeons proficiency description	Degree of proficiency	Passed learning curve
Hu	“performed by a single senior surgeon”	High	Yes
Siljander	“had completed more than 100 DA cases”	High	Yes
Wu	“easier to achieve femoral exposure than in general hips”	High	ND
Barrett	“had performed over 100 DAA cases”	High	Yes
Daas	“first half of 2012 and 2011 were excluded, introduced in our hospital in early 2011”	High	Yes
Fleischman	“cases performed during a surgeon’s learning curve were excluded”	High	Yes
Godoy-Monzon	“reducing the possibility of complications attributable to the learning curve”	High	Yes
Triantafyllopoulos	“have extensive experience and may be considered experts”	High	Yes
Lee	“hip arthroplasty fellowship in both the PLA and the DAA”	High	Yes
Rykov	“far beyond the learning curve of the DAA (> 200)”	High	Yes
Zhao	“the first 100 patients” “were not enrolled in the current trial”	High	Yes
Fransen	“had performed 120 PLA and 80 DAA”	High	Yes
Jelsma	“ surgeon is using his own approach in which they trust”	High	ND
Langlois	“undergoing their subspecialty training” “equivalent to registrars”	High	ND
Amlie	“Patients registered before 2011” “were excluded”	High	Yes
Barrett	“with over 3000 PA cases vs 100 DAA cases”	High	Yes
Nam	“perform more than 200 THAs annually”	High	Yes
Spaans	“surgeons had an internal education” “who had used the DAA for 5 years”	High	Yes

*ND* unclear description

### Limitations

Since there was no blind method of participants and personal, most of the literatures has a high risk of bias, so subjective impressions can affect the results. Publication bias exists in this study; however, the degree of bias was acceptable. DAA is a surgical procedure that has only been developed in recent years, so it is not widely used, but it can be learned and mastered. The accuracy of the study may have been affected by the fact that a small number of researchers did not specify in the paper that the surgeon had passed the learning curve, and that the number of patients included in the RCT literatures were less than 50.

## Conclusion

The study showed that DAA was superior to the PLA after THA in regards to reducing length of hospital stay, LLD, PE/DVT, dislocation. Postoperative pain was mild, and the recovery of hip function was faster and earlier. Thus, DAA may be a better option for patients with hip disease requiring THA. In consideration of the limitations of this study, we need more randomized controlled trials to compare the clinical outcomes of DAA with PLA.

## Data Availability

All data were contained in the text and charts of published articles.

## Abbreviations

DAA: Direct anterior approach
PLA: Posterolateral approach
THA: Total hip arthroplasty
RCT: Randomized controlled trial
WMD: Weighted mean difference
CI: Confidence interval
OR: Odds ratio
BMI: Body mass index
PRISMA: Preferred Reporting Items for Systematic Reviews and Meta-analyses
VAS: Visual analog scale
HHS: Harris hip score
HOOS: Hip disability and osteoarthritis outcome
PE/DVT: Pulmonary embolism/deep vein thrombosis
LLD: Leg-length difference
