# Correction to: Direct anterior versus posterolateral approaches for clinical outcomes after total hip arthroplasty: a systematic review and meta-analysis

**DOI:** 10.1186/s13018-020-01893-2

**Published:** 2020-09-15

**Authors:** Wang Chen, Jian-Ning Sun, Ye Zhang, Yu Zhang, Xiang-Yang Chen, Shuo Feng

**Affiliations:** Department of Orthopedic Surgery, Affiliated Hospital of Xuzhou Medical University, 99 Huaihai Road, Xuzhou, 221002 Jiangsu China

**Correction to: J Orthop Surg Res (2020) 15:231**

**https://doi.org/10.1186/s13018-020-01747-x**

Following publication of the original article [1], we have been informed that there are incorrect statements in results section.

Corrected version:

In the ***Outcomes-Fracture*** section, “and the results obtained can prove that **PLA** has a higher fracture rate in patients than **DAA**” should be corrected to “and the results obtained can prove that **DAA** has a higher fracture rate in patients than **PLA**”.

In the ***Outcomes-Dislocation*** section, “showing **PLA** has a lower hip dislocation rate than **DAA**” should be corrected to “showing **DAA** has a lower hip dislocation rate than **PLA**”. Other contents need not be corrected after inspection.
